# Robot-assisted, CT-guided placement of responsive neurostimulator system with bilateral centromedian thalamus depth electrodes for multifocal intractable epilepsy

**DOI:** 10.3171/2024.4.FOCVID243

**Published:** 2024-07-01

**Authors:** Hael Abdulrazeq, Josh Feler, Anna R. Kimata, Wael F. Asaad, Athar N. Malik

**Affiliations:** 1 Department of Neurosurgery, Rhode Island Hospital, Providence;; 2 The Warren Alpert Medical School of Brown University, Providence;; 3 Norman Prince Neurosciences Institute, Rhode Island Hospital, Providence; and; 4 Department of Neuroscience, Brown University, Providence, Rhode Island

**Keywords:** responsive neurostimulation, epilepsy surgery, robot-assisted surgery, stereotactic neurosurgery

## Abstract

The responsive neurostimulator system has become increasingly popular in the surgical management of refractory epilepsy, with targeting of various thalamic nuclei showing promising results in select patients. A 42-year-old female presented for evaluation of refractory epilepsy consisting of generalized tonic-clonic and focal seizures with preserved awareness. Phase I and II monitoring suggested multifocal bilateral epilepsy with bilateral frontal onset, and the patient underwent robot-guided bilateral centromedian thalamic placement of the RNS System. In this operative video, the authors share their institutional experience and protocol utilizing the ExcelsiusGPS robot in the placement of the RNS System in the thalamus.

The video can be found here: https://stream.cadmore.media/r10.3171/2024.4.FOCVID243

## Transcript

### 0:24 Case History.

A 42-year-old right-handed female with history of relapsing remitting multiple sclerosis presents for evaluation of medication refractory epilepsy. She was diagnosed at age 31, and seizure frequency had been increasing since age 40. Her seizure semiology consisted of generalized tonic-clonic seizures every few weeks, as well as focal seizures with rightward head turn and eye deviation with preserved awareness every few months. Current medications include clobazam, lacosamide, lamotrigine, and levetiracetam. She is neurologically intact on exam.

### 0:52 Preoperative Imaging.

A structural and functional MRI of the brain was obtained which revealed multiple demyelinating lesions apparent on the axial T2 MRI, and bilateral hippocampal atrophy. She was noted to have left-sided language dominance, and video-EEG had no clear localizing or lateralizing features.

### 1:10 Case History.

The case was discussed at the comprehensive epilepsy conference, and she was recommended for bilateral stereotactic EEG placement. She was then admitted to the epilepsy monitoring unit for 10 days. Data collected revealed multifocal bilateral epilepsy with predominantly bilateral frontal onset, and based on this the patient was recommended for bilateral centromedian thalamus implantation of responsive neurostimulator system.

### 1:35 NeuroPace RNS System Background.

After its approval by the FDA for adults with medically intractable partial-onset seizures, the NeuroPace RNS System has become increasingly popular in the treatment of a wide variety of epilepsies.^1,2^ RNS offers certain advantages over DBS, including its capacity to record data from ictal events, which can help further characterize patients’ seizure networks. RNS also offers adaptive stimulation and flexibility in modulating different control points in a patient’s seizure network. The thalamus in particular has been studied as a target for neuromodulation given its importance as a relay node for epileptic networks.^3–8^ The SANTE trial was a landmark point showing promising results for deep brain stimulation (DBS) targeting the anterior nucleus of the thalamus, particularly in patients with seizures originating from the temporal lobes.^4^ Given its projections to motor and premotor frontal regions, the centromedian thalamus has also been considered a viable option and studied in patients with multifocal epilepsy with predominantly frontal origin.^3,9^

### 2:40 Surgical Planning.

MRI with FGATIR sequence as well as CT angiogram are obtained preoperatively for planning purposes and uploaded to the Globus ExcelsiusGPS planning software. The ExcelsiusGPS robot offers both cranial and spinal surgical applications, with an integrated navigated and robotic trajectory alignment in one system. Other systems can be used depending on what is available at the institution. We use its software to determine the entry site as well as the target and trajectory. The entry site is just anterior to the coronal suture for this target. A combination of indirect targeting with AC-PC coordinates, as well as direct targeting using anatomical landmarks on the FGATIR sequence, are utilized. In this case, on the right side, our depth to target was about 8 mm right of the midcommissural point, 13 mm posterior, and 1 mm superior. This target will represent the tip of the lead, with the trajectory and lead contacts passing through the actual location of CMT, which appears hypointense on the FGATIR. The trajectory is chosen such that it is not transventricular and avoids any apparent vessels on the CTA.

### 3:50 Surgical Setup.

The patient is brought into the OR and intubated in standard fashion by the anesthesia team. A complete or minimal head shave can be performed depending on patient preference. Head can be clamped with the Mayfield frame, but here we chose the Leksell frame considering the target and lead placement as well as IPG location. The Leksell is applied in a standard fashion with anterior pins a fingerbreadth above the eyebrows and the posterior pins above the nuchal line. We ensure that the pins are finger tight, and then the frame is attached to the Mayfield attachment onto the bed. All pressure points are padded appropriately, and the patient’s arms are tucked bilaterally, and they are kept in the supine position as such.

### 4:27 Surgical Setup.

The frame-based reference array is then attached to the Leksell frame, which will be the reference for navigation with the robot during the case, and the intraoperative CT (ICT) array is attached to the Leksell frame such that it is parallel to the reference frame and as close to the scalp at the operative site as possible.

### 4:42 Surgical Setup.

An intraop CT is then obtained with the Airo scanner (this can be performed with the O-arm or Ziehm depending on resources available) and the anatomical landmarks are checked. The ICT frame then can then be removed as well as the nonsterile navigation spheres on the frame. The scalp is prepped and draped widely in the standard fashion, and sterile navigation spheres are attached through the drapes.

### 5:05 Surgical Video: Incision, Burr Holes, Lead Placement.

The robot arm is then brought into the field, and a stylet is inserted through the end effector to mark the entry site for each lead. The incisions for the entry sites are marked with a marking pen.

We infiltrate the incisions with lidocaine with epinephrine. We then use the IPG template to mark our incision for the flap of the IPG. This location is chosen so that it is a well-vascularized flap, and cosmetically favorable and accessible to the patient to interact with the device and swipe the magnet when using it. A 10 blade is used to open the incision down to the skull. This is carried down with the Bovie. We then use a periosteal dissector to reflect the periosteum off the skull. Hemostasis is achieved with a bipolar cautery. The end effector is then brought in again, and we insert the stylet to ensure that we have adequate exposure for our burr holes. A perforating drill is then used to create the burr holes bilaterally. Bone wax is used for hemostasis as well as to prevent air embolism through venous lakes in the skull, especially given the somewhat elevated head position here. The robot’s end effector is then brought into target, and we ensure that the stylet enters the burr hole without any deflection. Excess bone wax is removed with a curette.

The round cutting burr is used to countersink the location of the cover device that will secure the RNS leads, and we ensure that it sits perfectly within this countersink and the screws are tightened. A cranial plating system may be used instead to secure the leads.

We measure the distance from the outer cortex of the skull to the target, and we mark this on the leads, as well as the distance from the end effector to target, and the distance from the top of the slotted cannula that will be used to insert the leads down to target.

We now open the dura with an 11 blade in cruciate fashion and use bipolar cautery to achieve hemostasis of the dural leaflets as well as create our corticectomy.

We bring the robot back into the field again, and now we are ready to insert a slotted cannula with a stylet down to target after ensuring that the patient has no elevation in blood pressure. We then bring the stylet out of the cannula and insert the lead. We check that we have inserted the lead down to the marks that we made earlier. Now we remove the slotted cannula over the lead ensuring that it does not move. A piece of Gelfoam can be placed at the entry site.

We place the cover for the burr hole cover device around the lead and lock it in place. The stylet is removed from the lead; then using bayoneted forceps or rubber shod clamps, we remove the leads from the end effector. A mark is made with a pen at the lead’s exit point at the cover device to ensure it does not move for the remainder of the case, and a protective boot is used to cover the end of the lead.

### 8:16 Intraoperative Imaging.

An intraop CT can then obtained after both leads have been placed. We upload this CT to the robot and merge with the preoperative MRI to check the placement with the planned trajectory to ensure accuracy.

### 8:30 Surgical Video: Implantable Pulse Generator Placement.

Now we are ready to open the flap for the IPG. We first use a 10 blade, and Raney clips are applied to achieve hemostasis from the scalp edges. A periosteal is used to dissect the periosteum off. Now we bring the template and mark the edges of it and we are ready to start performing our craniectomy with a perforating drill. The burr hole is created at the location of the set screw on the IPG tray. A Woodson dental is used to dissect the dura off the inner table of the skull. The footed craniotome attachment is used to complete our craniectomy. The craniectomy must be close to the size of the tray to ensure that it can be secured to the skull edge. The flap is removed carefully, and hemostasis is achieved with bipolar cautery for the dura. The gutters are lined with Floseal to ensure that we are minimizing the risk of an epidural hematoma. Some surgeons may place a single layer of Surgicel between the dura and the tray. The tray for the neurostimulator is then put in place and secured with 4-mm screws to the skull. Now the IPG itself can be put in place and secured to the tray.

We tunnel the left-sided lead to the right-sided incision, and then we tunnel both leads through the right side and the leads are then secured to the IPG.

### 9:46 Surgical Video: ECoG and Closure.

Now we are able to check live electrocorticographies intraoperatively to ensure that we are obtaining good signal and that impedances are okay. We create a strain loop for the leads and ensure that they are not on top of the generator or under the incision line. We irrigate the wounds with vancomycin irrigation and place vancomycin powder, and now we close all the wounds in layers with 2-0 Vicryl for the galea and staples for the skin. Bacitracin ointment and Xeroform and a sterile dressing are applied.

## Disclosures

Dr. Kimata reported shareholder in NeuroPace outside the submitted work.

## Author Contributions

Primary surgeon: Malik. Assistant surgeon: Feler. Editing and drafting the video and abstract: all authors. Critically revising the work: all authors. Reviewed submitted version of the work: all authors. Approved the final version of the work on behalf of all authors: Abdulrazeq. Supervision: Abdulrazeq, Asaad, Malik.

## Supplemental Information

### Patient Informed Consent

The necessary patient informed consent was obtained in this study.
